# The Role of miRNA in Papillary Thyroid Cancer in the Context of miRNA Let-7 Family

**DOI:** 10.3390/ijms17060909

**Published:** 2016-06-15

**Authors:** Ewelina Perdas, Robert Stawski, Dariusz Nowak, Maria Zubrzycka

**Affiliations:** 1 Department of Experimental Physiology, Chair of Experimental and Clinical Physiology, Medical University of Lodz, Lodz 92-215, Poland; maria.pawelska-zubrzycka@umed.lodz.pl; 2 Department of Clinical Physiology, Medical University of Lodz, Lodz 92-215, Poland; robert.stawski@umed.lodz.pl (R.S.); dariusz.nowak@umed.lodz.pl (D.N.)

**Keywords:** miRNAs, let-7 family, papillary thyroid cancer, carcinogenesis

## Abstract

Papillary thyroid carcinoma (PTC) is the most common endocrine malignancy. RET/PTC rearrangement is the most common genetic modification identified in this category of cancer, increasing proliferation and dedifferentiation by the activation of the RET/PTC-RAS-BRAF-MAPK-ERK signaling pathway. Recently, let-7 miRNA was found to reduce RAS levels, acting as a tumor suppressor gene. Circulating miRNA profiles of the let-7 family may be used as novel noninvasive diagnostic, prognostic, treatment and surveillance markers for PTC.

## 1. Introduction

Thyroid cancer is the most widespread endocrine malignancy. There are four main varieties of thyroid cancer: papillary (PTC), follicular (FTC), anaplastic (ATC) and medullary thyroid cancer (MTC). PTC, FTC and ATC originate from follicular cells and only MTC originates from parafollicular cells (C-cells). More than 95% of thyroid cancers derive from follicular thyroid cells. PTC is the most common and accounts for approximately 80%–90% of human thyroid cancers [1,2,3,4].

MicroRNAs (miRNAs) are endogenous, single-stranded, noncoding 18- to 25-nucleotide RNAs. miRNAs are involved in many biological and pathological processes: proliferation, differentiation and apoptosis. miRNAs regulate gene expression at the post-transcriptional level of messenger RNAs (mRNAs) by selectively binding to the matching 3-untranslated region (3-UTR) of the target mRNA through base pairing and they prevent the translation process by destroying or blocking the mRNA. Some studies have suggested that miRNAs might bind to the 5′-UTR or open reading frame (ORF) of the target mRNA [5,6,7].

There are two main ways in which miRNAs could contribute to cancer development. The first way is the upregulation of some miRNAs which could lead to the silencing of tumor suppressor genes. Alternatively, miRNA downregulation could result in increased oncogene expression. Deregulation of specific miRNA could lead to the decreased expression of tumor suppressor genes and/or the increase of oncogene expression [8].

miRNA expression profiles are different not only between tumors and normal tissues but also between different types of tumors, between different stages of the same tumor and between primary tumors and metastatic tumors [7].

Let-7 was firstly known as a factor promoting differentiation in *C. elegans* (in the transition from the larval to adult stage) and since then has been confirmed as a key regulator of gene expression in many organisms. The let-7 family (let-7a, let-7b, let-7c, let-7d, let-7e, let-7f, let-7g and let-7i) is a well-known miRNA and is an important tumor suppressor in various cancers. The let-7 family shows high sequence and function conservation across multiple species. Family genes of let-7 are located on different chromosomes and are one of the most expressed miRNAs in normal thyroid glands, also suggesting its important role in typical thyroid development and functionality. In all thyroid carcinomas originating from follicular cells, the expression level of the let-7 family is decreased [2,9,10,11].

Some data have indicated the opportunity to use circulating miRNAs (plasma, serum or other body fluids) as diagnostic, prognostic and predictive biomarkers in cancer. Normal tissue and cancers are different in expression profiles of circulating miRNAs [5,12].

Furthermore, circulating miRNAs have the benefit of being stable molecules because they are not sensitive to RNase activity. This feature of miRNAs can be useful in noninvasive techniques [5].

In this review, we briefly summarize the present state of knowledge about the let-7 miRNA family and its biological functions in papillary thyroid cancer.

Additionally, we present current scientific knowledge on the involvement of the let-7 family in the development of PTC.

## 2. Biosynthesis of miRNA

**Figure 1:**
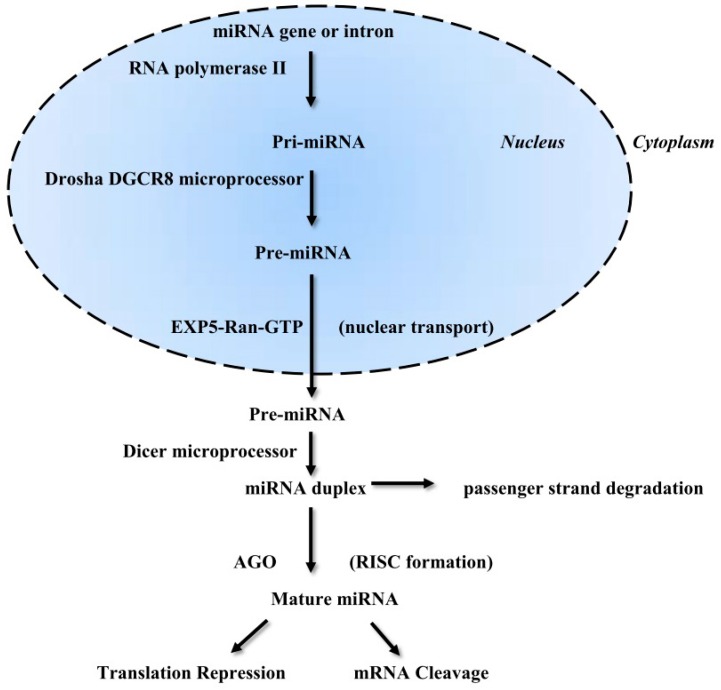
MicroRNA maturation and function. RNA polymerase II synthesizes primary miRNA. Next, pri-miRNA is processed by the Drosha DGCR8 microprocessor in the nucleus. The produced pre-miRNA is transported to the cytoplasm with the participation of EXP5-Ran-GTP. In the next step, the Dicer microprocessor creates a mature miRNA from pri-miRNA by removing the loop. The mature miRNA with AGO protein forms RISC to stop the translation of a target mRNA.

RNA polymerase II produces a primary miRNA (pri-miRNA) transcript with a 5′ cap and a 3′ poly(A) tail. Internal base-pairing inside pri-miRNA creates a specific hairpin stem-loop structure with a stem of ~33 base pairs (bp) in length. In the nucleus, pri-miRNA is processed by microprocessor complex Drosha (RNase III enzyme) and DGCR8 (DiGeorge syndrome critical region 8), also known as Pasha, to form 60- to 100-nucleotide-long precursor miRNA (pre-miRNA). The Drosha/DGCR8 microprocessor is a heterotrimeric complex consisting of one Drosha and two DGCR8 proteins. The Ran-GTP-dependent transporter Exportin-5 (EXP5) was described to support the transport of pre-miRNAs from the nucleus through the nuclear pore complex to the cytoplasm. When the pre-miRNA/EXP5/Ran-GTP complex is transferred to the cytoplasm, GTP is hydrolyzed to GDP and the pre-miRNA consequently dissociates. In the cytoplasm, pre-miRNAs are processed into 22–25 nucleotide miRNA duplexes with the participation of the cytoplasmic Dicer (RNase III enzyme) by removing the loop. In the duplex, one strand is the guide strand, and the second complementary strand is the passenger strand. The guide strand is loaded onto an Argonaute (AGO) protein to form RISC (RNA-induced silencing complex), which recognizes a target sequence and the passenger strand is degraded. The mature miRNA-RISC complex identifies its matching sequences in the 3′ UTR of their target mRNAs by seed region (usually positions 2–7 in the miRNA). RISC-incorporated mature miRNAs can obstruct gene expression through post-transcriptional actions, such as stopping translation or supporting mRNA degradation. A single miRNA can control various genes and a single gene can be regulated by several miRNAs (Figure 1) [3,5,7,12,13,14,15,16,17].

## 3. Lethat-7 (Let-7) Family in PTC

The most common genetic alterations in PTCs include RAS mutation, BRAF mutation, and RET/PTC rearrangement which are mainly involved in the RET/PTC-RAS-BRAF-MAPK-ERK signaling pathway. Importantly, many studies have proved that BRAF mutation is related to high-risk clinicopathological features, such as larger tumor size, extrathyroidal extension, local lymph node metastasis, long-distant metastasis, and progressive disease stages, signifying that it is a self-regulating oncogenic occurrence for PTC tumorigenesis and is involved in the progression of PTC [3,7,18].

Let-7 was shown to have complementary binding sites in the 3′-UTR of all three RAS genes (HRAS, KRAS, and NRAS), and it functions to decrease RAS protein levels. Because RAS activation is related with several cancers, let-7 downregulation or deletion could be crucial in tumorigenesis [14,18,19].

Downregulation of some members of the let-7 family is observed in PTC. For example, let-7f miRNA has lately been linked with RAS protein level development in PTC. Decreased expression of let-7f has been shown in independent studies by Pallante *et al.*, Visone *et al.* and Braun *et al.* via microarray in PTC samples [20,21,22]. Moreover, in thyroid cell lines with RET mutation, decreased expression of let-7f is linked with the increased expression of the RET oncogene. The TPC-1 cell line with RET mutation stably transfected with a vector causing the overexpression of let-7f suppressed MAPK activation and restricted cell proliferation. Furthermore, let-7f enhances the expression of the TITF1 transcription factor–thyroid cell differentiation markers, a crucial element in typical thyroid development. Therefore, let-7f is essential for the precise control of thyroid cell growth and development. Furthermore, decreased expression of let-7f in a thyroid cell line with RET mutation is suspected of being responsible for cell dedifferentiation during PTC malignant progression [18]. This data suggested let-7f could play an important role as a tumor suppressor and designated this miRNA as a possible therapeutic aim in patients with PTCs with a RET mutation [5]. Furthermore, Braun *et al.* identified a decreased expression of let-7g. In opposition, Schwertheim *et al.* showed an increased expression of let-7c in PTCs (nine analyzed samples) compared to non-tumorous tissue [23]. Overexpression of let-7c in the PTCs had already been shown by Viston *et al.* in 2007 via microarray. This was confirmed by Braun *et al.* in 2010 and the results were validated with TaqMan MicroRNA assay. Moreover, Cahill *et al.* examined the expression profile of miRNA in two RET-mutated human PTC cell lines, compared to normal thyroid cell lines, discovering significantly overexpressed levels of let-7d [24]. However, Braun *et al.* and Swierniak *et al.* identified a decreased expression of let-7d in PTC samples [22,25]. Gao *et al.* identified an increased expression of let-7b in the invasive subpopulations compared to the control subpopulations of PTC cells [26]. Damanakis *et al.* in 2015 identified an increased expression of let-7b in 12 out of 14 analyzed samples of PTCs. Furthermore, a group of patients with PTC was distinguished by a significant overexpression of let-7f (about 100-fold, two times up to 10^6^-fold) compared to normal thyroid tissue. In addition, the authors analyzed the expression of HMGA2 (high mobility group AT-hook 2) and the SLC5A5 gene. HMGA2 is typically expressed during normal embryogenesis and tissue development. Generally, the gene is not expressed in differentiated tissue. HMGA2 protein has three basic DNA-binding domains (AT-hooks) and could function as a transcriptional regulating factor, and it may be related to the abnormal growth of the human thyroid. However, the results showed that the expression of HMGA2 is not correlated with the expression of the let-7 family. Interestingly, the expression of SLC5A5 was decreased in PTC and its expression could be regulated by let-7f. Dysfunction of the sodium-iodide symporter (NIS) may cause resistance to radioiodine. The reason for this is usually suppression or loss of the NIS gene (SLC5A5). [27]. This let-7 family member is reported to play an important role in tumor cell metastasis progression. All the described miRNAs are presented in Table 1.

## 4. Expression Profiles of Circulating miRNA in PTC

miRNAs from the cell can be released into the nearby microenvironment membrane or the circulation-free, protein-bound, associated with HDL or packaged within microvesicles (0.1–1 µm) or nanovesicles (<100 nm). Free miRNAs are quickly degraded by RNases, whereas vesicular, protein-bound miRNAs or those associated with HDL are protected from degradation. These circulating miRNAs released by the cells are presently being considered as possible noninvasive biomarkers of disease for recurrence or diagnosis. The stability of miRNAs in the circulation suggested that they could regulate gene expression in distant cells. Currently, the potential function of extracellular miRNAs is being studied intensively. Some studies have confirmed that miRNAs may indeed function in the communication between cells. Some of these circulating miRNA biomarkers could be useful as prognostic factors for survival, extracellular communicators, staging tools, and markers of pathological progression in various cancer types. Previous studies have proved that circulating levels of let-7e are higher in PTC patients compared to positive controls (healthy subjects). Yu *et al.*, using Solexa sequencing followed by extensive qRT-PCR, examined miRNA expression in the serum of 245 subjects (106 patients with PTC, 95 patients with benign nodules, and 44 controls) [29]. However, there were no significant differences in the serum levels of miRNAs in the group of patients with benign nodules compared with the positive controls. Interestingly, they found that serum levels of let-7e were statistically significantly increased in PTC patients compared with patients with benign nodules and the positive controls. Moreover, Li *et al.* demonstrated that the levels of plasma let-7i were significantly higher in the PTC group compared with the benign nodules group based on microarray. Further validation by quantitative RT-PCR indicated that the levels of plasma let-7i were significantly higher in the PTC group than in those with benign nodules or the positive controls [30]. Moreover, Graham *et al.* in 2015 identified an increased expression of serum let-7b both in benign and PTC patients, and presented a statistically significant increase when they were compared [28]. Let-7b is often significantly overexpressed in various cancers, and also could cause overexpression of oncogenes (e.g., Ras, c-Myc and MDM4). Raised let-7b expression in PTC may have a similar effect on these genes (Table 1). Therefore, it appears that overexpression of the Ras oncogene caused a mutation, and thus the uncontrolled proliferation of thyroid cancer cells is linked with a decrease in the expression of the let-7 family. Furthermore, the overexpression of c-Myc is associated with enhanced cell growth in various cancers. The oncogene c-Myc is a crucial regulator of miRNA transcription, and interestingly, the suppressive let-7 family, but also the miR-26, -29, -30 and -34 families. Loss of the let-7 family could be a first step in the progression of cancer, uncontrolled tumor growth and metastasis at advanced stages [2]. This miRNA had a high diagnostic sensitivity and specificity for PTCs and its expression levels were correlated with certain clinicopathological features, such as tumor size, multifocal lesion status, nodal status, and the TMN (tumor metastases nodules) stage.

A number of studies have reported that circulating let-7 is downregulated in numerous types of cancer. The levels of circulating let-7b were statistically significantly decreased in women with endometriosis matched with healthy subjects. Interestingly, let-7d and 7f showed a tendency toward downregulation. In breast cancer patients, the levels of let-7a were expressed at a higher level in the circulating blood than in the controls. Interestingly, the levels of miRNA decreased significantly after curative tumor resection. A low level of let-7 is significantly correlated with a shorter survival time after surgery in patients with lung cancer. The let-7 miRNA family is a crucial agent in the proliferation, apoptosis and metastasis of cancer cells. The expression level of let-7 is potentially a link between oncogenes and their signaling pathways and the stage of the tumor. This proves that circulating miRNA can be an effective biomarker for various types of cancers [31,32,33,34].

## 5. Conclusions

miRNAs are important regulators of gene expression in many essential cellular processes such as proliferation, cell cycle control, signaling pathways and apoptosis. Incorrect changes in the expression of miRNA are involved in the initialization, development, and metastasis of human cancer. MiRNA expression profiles are different between tumors and normal tissues. Furthermore, miRNA expression levels are also different between tumors’ stages of malignancy, and between different histopathological lesions of the same tissue. In addition, profiles of miRNAs are different in the case of the primary tumor and metastasis. Current results suggested that miRNA expression profiles could be a useful tool for classifying poorly differentiated human tumors that cannot be precisely categorized only by the usual mRNA expression profiles. Moreover, in the case of tumors, diagnostic and histopathological characterization expression profiles of miRNA may be used as biomarkers. Furthermore, the therapeutic management of patients could be supported by the examination of miRNA expression profiles; differences in miRNA expression profiles are associated with the future outcomes of therapy.

Additionally, miRNAs could be promising targets for gene therapy for the prevention of the initiation and progression of human cancer. miRNAs could be targets for therapy based on RNA use, both positively modifying the expression of miRNAs *in vivo* and/or inhibiting miRNA expression.

## Figures and Tables

**Table 1:** miRNA characteristics of papillary thyroid cancer. TPC-1 human PTC cell line spontaneously harboring the RET/PTC1 rearrangement. IHH-4 human papillary thyroid cancer cell line.

miRNA Name	Up/Downregulation	Specimen Type	Reference
Let-7b	Up↑	Cell line IHH-4	[26]
	Up↑	Thyroid sample	[27]
	Up↑	Serum (circulating)	[28]
Let-7c	Up↑	Thyroid sample	[23]
	Up↑	Thyroid sample	[21]
Let-7d	Up↑	Cell line TPC-1	[24]
	Down↓	Thyroid sample	[22]
	Down↓	Thyroid sample	[25]
Let-7e	Up↑	Serum (circulating)	[29]
Let-7f	Down↓	Cell line TPC-1	[18]
	Down↓	Thyroid sample	[20]
	Down↓	Thyroid sample	[21]
	Down↓	Thyroid sample	[22]
	Up↑	Thyroid sample	[27]
Let-7g	Down↓	Thyroid sample	[22]
Let-7i	Up↑	Plasma (circulating)	[30]

## Abbreviations

3-UTR	3-untranslated region
AGO	argonaute
ATC	anaplastic thyroid cancer
DGCR8	diGeorge syndrome critical region 8
ERK	extracellular regulated kinase
EXP5	exportin5
FTC	follicular thyroid cancer
HMGA2	high mobility group AT-hook 2
Let-7	lethal-7
MAPK	mitogen-activated protein kinase
miRNA	micro ribonucleic acid
mRNA	messenger RNAs
MTC	medullary thyroid cancer
ORF	open reading frame
PI3K	phosphatidylinositol-3-kinase
pre-miRNA	precursor miRNA
pri-miRNA	primary miRNA
PTC	papillary thyroid cancer
RET/PTC	rearranged in transformation/papillary thyroid carcinoma
RISC	RNA-induced silencing complex
TMN	tumor metastases nodules
